# Green synthesis of iron nanoparticles to promote seed germination of *Zea mays* under salinity condition

**DOI:** 10.1016/j.heliyon.2025.e41823

**Published:** 2025-01-09

**Authors:** Hiba Fouad Abdulfatah, May Fahmi Abdulrahman, Enas Fahd Naji

**Affiliations:** a Lec., Department of Biology, College of Sciences, University of Anbar, Al-Anbar, Iraq; b Lec., Department of Applied chemistry, Applied Science Colloge- Hit, University of Anbar, Al-Anbar, Iraq

**Keywords:** Root length, Maize, Salinity stress, Iron nanoparticles, Green synthesis

## Abstract

The application of green synthesis of nanoparticles in agriculture serves as an environmentally benign strategy. Pomegranate Peel extract (PPE) was utilized to produce iron nanoparticles (Fe NPs). PPE contains biomaterials that have the ability to synthesize Fe NPs through the reduction the iron precursor salt (FeSO_4_•7H_2_O) and also act as capping and stabilizing agents. UV–Visible, FTIR, XRD, SEM, EDX, and nano zeta-potential analysis were used to investigate Fe NPs. The synthesis of Fe NPs was confirmed by a significant color change from yellow to black. Then Fe NP production is confirmed by a UV–Visible peak at 301 nm. FTIR showed O–H and C=C stretching due to phenol and alkene functional groups. X-ray diffraction showed that Fe NPs are mostly amorphous. The synthesized Fe NPs were spherical and have around 12 nm in size, with a 5.5 mV value, the synthesized Fe NPs were stable.

The effects of these (Fe NPs) on the germination of maize (*Zea mays* L.) labeled Z1(Buhooth 5018), Z2(Baghdad-3) and Z3 (Fajr-1) seeds and the growth of their roots were assessed under conditions of salinity at different levels (0, 8, 12 ds m^−1^) in a laboratory-scale system. The findings indicated that the application of the synthesized Fe NPs at a concentration of 20 Mm caused an increase in germination ratio, root and shoot length, seedling vigor index, root length stress tolerance index (RLSTI) and shoot length stress tolerance index (SLSTI). The findings showed that the stress-relieving benefits of Fe NPs were more effective than normal forms, which may be related to their distribution, shape, size, and other properties. In general, the results of the present research indicate that utilizing pomegranate peel extract for eco-friendly production of nanoparticles could enhance the germination of seeds and the strength of seedlings in *Zea mays*.

## 1 Introduction

Nanotechnology, with all its science and techniques, is experiencing fast development and growth covering a diverse collection of applications in biology, medicine, and engineering. Nanotechnology focuses on the production of nanoparticles with various sizes and shapes, as well as their potential applications [1]. Nanoparticles (NPs) are particulate structures with dimensions ranging from 1 to 100 nm, with many notable unique properties, such as a high surface-to-volume ratio, increased stability, minimized agglomeration, catalytic activity, and tunable size, as well as a wide range of application [2]. Microorganisms and plant materials can be used as a way out of conventional methods of physically or chemically synthesizing nanoparticles [3]. It has been seen that the idea of using naturally available resources for the production of metal nanoparticles is both cost-effective and eco-friendly [4]. Generally, plant extracts possess polyol components that are involved in bio reducing of metal ions during green synthesis process.

Moreover, water soluble heterocyclic components stabilize the resulting metallic particles [5]. Recent studies have paid increased attention to metallic nanoparticles (NPs) due to their beneficial biological properties, lack of toxicity, and exceptional features. Besides, their application in many sectors like water treatment [[6], [7], [8], [9]] as well as in pharmacology field [10]. Green synthesis strategies have recently shown potentiality in synthesizing iron nanoparticles (Fe NPs) with small sizes, large surface areas, and high biocompatibility [11]. These nanoparticles have shown great promise in the field of agriculture due to their ability to improve nutrient uptake and enhance plant growth [[12], [13], [14]].

Maize, a salt-sensitive crop, is a major food and fodder crop [15]. Drought, floods, heavy metals, and other stressors diminish maize productivity, but salt stress is linked to them [16]. Nanotechnology, an exceptionally innovative and efficacious discipline, has yielded truly remarkable outcomes within this field [17,18]. A pre-sowing process known as "seed priming" results in a Physiological modification in the seed enables faster germination [19,20]. Up to this point, nano-priming approaches have been employed to enhance seed germination, increase plant resistance to diseases, improve tolerance to stress, and boost production. Furthermore, this method offers a high level of control, in contrast to the use of nanomaterials through spraying on leaves. Therefore, nano-priming can be regarded as a more ecologically sustainable and less harmful to plants. Green synthetic nanomaterials can enhance the safety of nano-priming processes by reducing environmental risks and mitigating concerns about human toxicity associated with nanoparticles [21].

The current investigation involved the production of iron nanoparticles (Fe NPs) by a fast and efficient biosynthetic process. This method utilized an aqueous extract derived from the pomegranate peel extracts as both the reducing and capping agent. Further, the synthesized nanoparticles were evaluated in vitro for their effectiveness in enhancing growth and mitigate stress in maize seeds treated with different salinity levels was assessed. Our results support the potential application of Fe NPs for agricultural purposes as a sustainable method to boost plant productivity, aiding the faster and healthier growth of crops under saline conditions.

## 2 Material and methods

### 2.1 Chemical, reagents and pomegranate peel (PPE) collection

From a private farm near Hit, we obtained fresh pomegranate fruits (*P*. *granatum* L.), Al-Anbar, Iraq, during the summer season, 2022. Without further purification, all compounds were of analytical grade. Ferrous sulfate heptahydrate FeSO_4_.7H_2_O (99.5 %) were purchased from Sigma Aldrich. Distilled water (DW) was used wherever required.

### 2.2 Pomegranate peel extract (PPE) preparation

The collected pomegranate fruits were washed four times with tap water to remove any dust, dirt, or residues that might be present on the outer surface; followed by distilled water three times and peeled manually; the clean peels were open air dried at room temperature without any exposure to direct sunlight. Once the peels were properly dried, they were ground into a fine powder using an electrical grinder and stored at 4 °C until extraction. The pomegranate peel extract was made using an altered procedure by boiling 12 g of dried peel powder in 200 mL of distilled water at 60 °C for 30 min [22]. This produced an aqueous extract solution that was light yellow. After being allowed to cool to room temperature, the resultant solution was filtered using Whatman No. 2 filter paper and kept in the refrigerator to be used in the synthesis of the desired Fe NPs.

### 2.3 Formation of Fe NPs using pomegranate peel extract (PPE)

The yellow-colored peel extract was added dropwise to 20 mL aqueous solution of a freshly prepared (20 and 30 mM FeSO_4_. 7H_2_O) in 10:1 vol ratio with continuous stirring at room temperature. Subsequently, the solution temperature was increased and left at 60 °C for 5 min, then cooled to room temperature [23]. The formation of iron nanoparticles (Fe NPs) can be easily detected through a simple color change in the solution. In this case, the solution changed from yellow to an intense black, indicating the presence of Fe NPs.

### 2.4 Plant materials

#### 2.4.1 Seeds treatment and plant growth

This experiment was conducted in the laboratory of the college of science at the University of Anbar (March 2023) to study the effect of salt stress induced by different levels, namely 0, 8, and 12 ds m^−1^, on the germination and development of three Iraqi maize cultivars, Buhooth 5018, Baghdad-3, and Fajr-1 labeled Z1, Z2, and Z3 were obtained from the Center of Seeds Certification at Abu Ghraib city. The seeds were sterilized using a 10 % sodium hypo chloride solution for 2 min, then washed twice with distilled water and left to dry at room temperature. Sterilized seeds were subjected to nano-priming with (0, 20, and 30 mM) nanoparticles of Fe. Following a 24-h priming period with NPs, the seeds were thoroughly washed with distilled water and dried at 25 °C to roughly maintain their original weight.

These were maintained in the growth chamber for 10 days at 25 °C, 60 % relative humidity, with dark and light photoperiods.

#### 2.4.2 Statistics

The study utilized a complete randomized design (CRD) with three replicates for each saline treatment and thirty seeds for each concentration level, distributed among plastic containers with filter paper. In this research, GenStat 12th software was applied for data analysis.

### 2.5 Fe NPs ultraviolet absorption (UV–Vis) spectroscopy

To investigate the maximum wavelength of the localized surface plasmon resonance (LSPR), the as synthesized Fe NPs, after dispersion, were characterized by UV-1800, Shimadzu, UV–Visible spectrophotometer. The readout spectrum was recorded within the range of 200–800 nm to remark any alterations. These results offer certainty of validating the used synthesis method of effectively producing Fe NPs. Further, information regarding the size, shape, and other physical traits of the nanoparticles can be received from the spectrum of the SPR.

### 2.6 X-ray diffraction (XRD) and Fourier transform Infra-Red (FTIR) analysis of Fe NPs

Characterization techniques of X-ray Diffraction (XRD) and (FTIR) analysis was carried out for the powdered sample employing the KBr pellet technique in a (400-4000 cm^−1^) range for more understanding of structural characteristics.

### 2.7 Zeta potential of a synthesized Fe NPs

One important index that can characterize the surface electrostatic potentials of the particles and provide information on stability is the zeta potential. The magnitude of the zeta potential offers insight on the probable stability of the colloid. The key parameter that affect the long-term stability of formulated colloids is the zeta potential. The zeta potential measurements of Fe NPs were taken using a Malvern Zetasizer model Nano ZS.

### 2.8 Scanning electron microscopy (SEM) and (EDX) analysis of Fe NPs

For better insight regarding size and shape of the synthesized FE NPs, the samples were examined using a SEM instrument (TESCAN MIRA3 FRENCH). As well, to identify the composition of the Fe NPs, Energy-dispersive X-ray spectroscopy (XPS, Thermo Scientific Nexsa G2) was operated on the powdered samples.

## 3 Results and discussion

### 3.1 Characterization of Fe NPs

Due to nanoparticles properties that may largely impact their applications, particularly their size, shape, and morphology, nanoparticles have turned to bean a crucial choice for optimized and high potential applications in many fields, such as electronics, energy, and health and medicine.

**Fig. 1:**
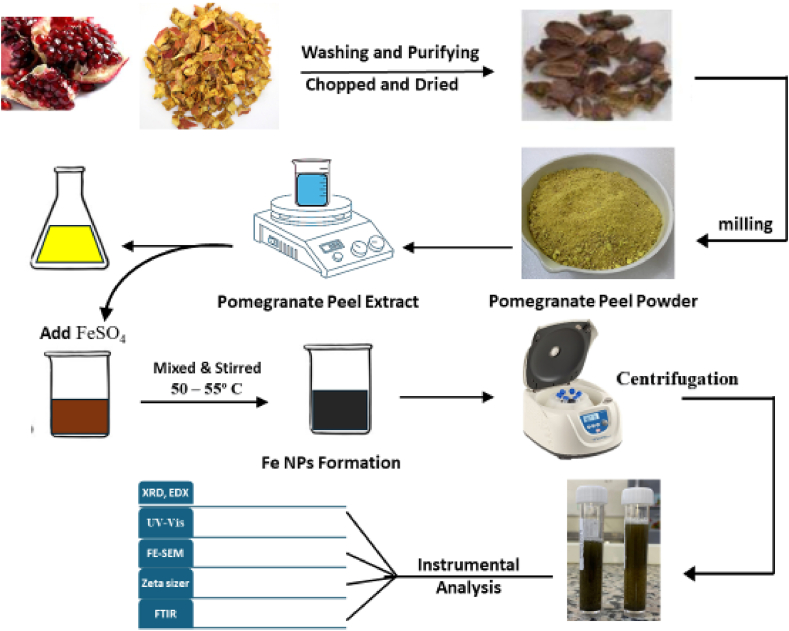
Synthesis route of synthesized iron nanoparticles Fe NPs using (PPE).

Several characterization techniques have been developed to have a greater understanding of these properties. Green synthesis of metallic nanoparticles using plant extracts is gaining importance in recent years because such plant materials are pharmacologically significant. This method has potential for the synthesis of metallic nanoparticles Fe NPs that serve for such purpose. In this research work, Fe NPs was prepared via a green route using the pomegranate peel PPE as shown in Fig. 1. The Fe NPs synthesis has been confirmed through a characteristic change in both the PPE and the aqueous FeSO_4_ mixture.

**Fig. 2:**
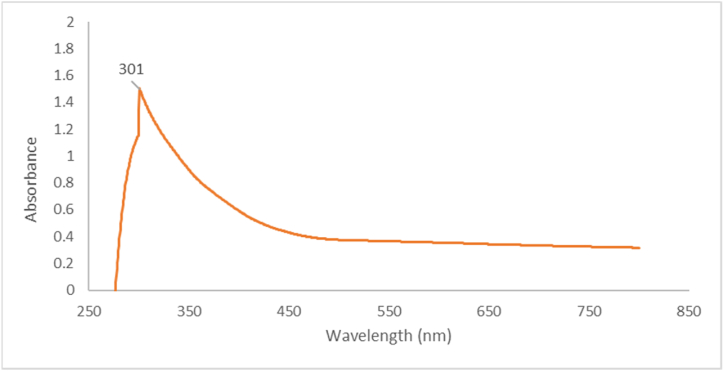
UV absorbance spectrum of synthesized iron nanoparticles Fe NPs.

The transparent yellow color of the mixture immediately turns black within seconds, indicating successful reduction of aqueous iron ions to Fe NPs when added to the PPE. The dark color is due to surface plasmon excitation vibrations in the Fe nanoparticles, which is further confirmed by the absorption bands around 301 nm (Fig. 2). Fe NP formation is a complex process that involves complexation of Fe salts followed by capping of Fe with phenolic compounds. These results are consistent with previously published research on Fe NPs synthesis [22].

**Fig. 3:**
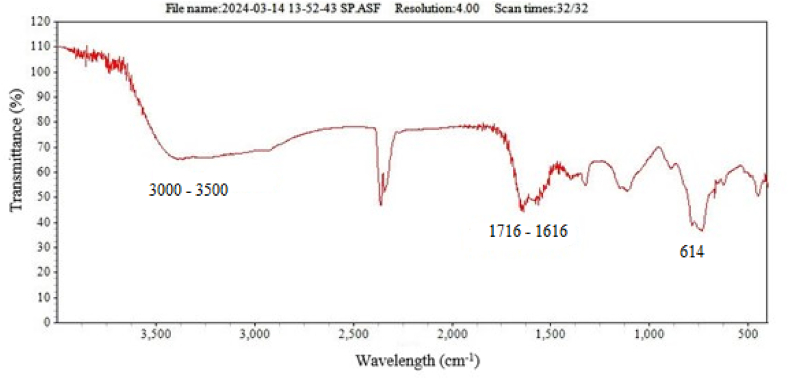
FTIR absorbance spectrum for synthesized iron nanoparticles Fe NPs.

FTIR analysis was used for to identify any biomolecules that may be responsible for the reducing of metal precursor ions and act as a stabilizing agent for Fe NPs nanoparticles. FTIR spectrum of the Fe NPs were collected in the range of 400–4000 cm^−1^, as shown in (Fig. 3). The obtained FTIR spectrum has a broad peak in the region of 3000–3500 cm^−1^ which represents various functional groups, such as alcohols, phenols (O–H stretch, H–bonded), carboxylic acids (O–H stretch), and amide groups (N–H stretching vibrations. The peaks observed at 1716-1616 cm^−1^ corresponded to the stretching of C=C amines (in-ring) and the C=C aromatic vibration, respectively. The presence of organic molecules on the surface of Fe NPs has been found to affect the FTIR peaks. The presence of Fe vibrations was responsible for the band observed at 614 cm^−1^ [24].

**Fig. 4:**
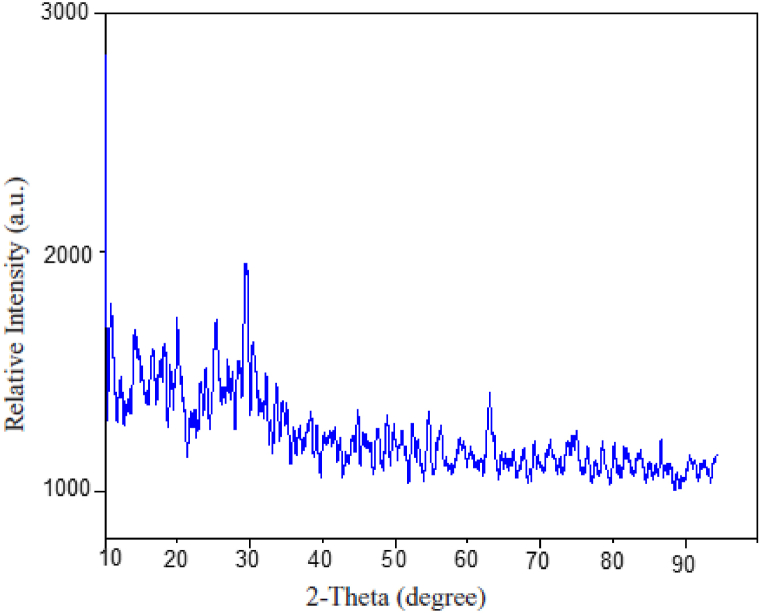
XRD pattern of synthesized iron nanoparticles Fe NPs.

Fig. 4 displays the XRD pattern of Fe NPs nanoparticles. The presence of nano crystalline iron peaks throughout the sample is confirmed (reference code: 96-403-8841). The abundance of peaks in this diagram also proves the formation of nano-iron in small crystal sizes. Yet, the main sharper peaks of higher intensity were 2θ of values (17.02, 28.04, 29.94) corresponding to crystal Fe NPs. Furthermore, smaller peaks of less intensity were observed overlapping through the graph indicating crystallinity of larger crystal sizes due to seem be aggregation and clumping of Fe NPs with each other. Such aggregation was notably seen in the figures of SEM. Nevertheless, the XRD pattern also has conspicuous peaks which can be attributed to the presence of organic substances adsorbed from fruit extract acting as a capping or stabilizing agent [25,26].

**Fig. 5:**
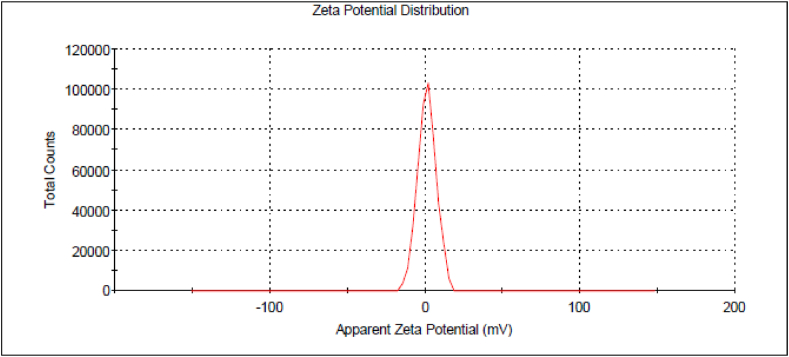
Zeta Potential (ZP) analysis of synthesized iron nanoparticles Fe NPs.

Zeta Potential (ZP) analysis was employed to verify the surface charge of NPs, which characterizes the electrical properties of the movement of NPs. The zeta potential degree indicates the amount of electrostatic repulsion between neighboring and similar charged particles in a dispersion. A nanosuspension exhibiting a zeta potential ranging from (+8.50 mV to −8.50 mV) is regarded as highly stable [27]. The zeta potential data observed for green synthesized Fe NPs are presented in the following Fig. 5. The Zeta potential of the Fe NPs was found to be 5.5 mV.

**Fig. 6:**
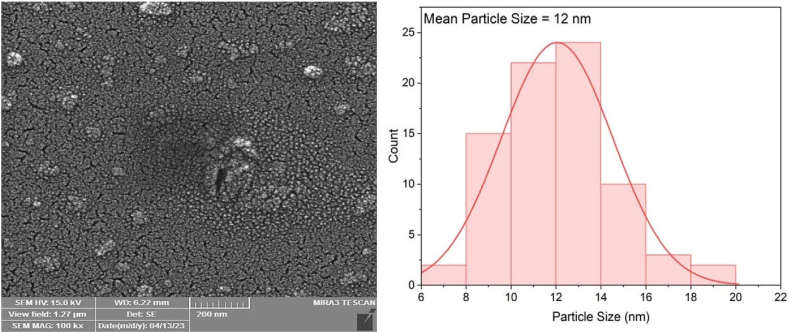
The morphology (FESEM images) and size distribution of synthesized iron nanoparticles.

Recently, there has been increasing interest in developing methods to produce nanoparticles with precise size and shape control. This is important because the properties of nanoparticles depend heavily on their size and shape, and by manipulating these factors, scientists can customize the properties of nanoparticles for particular uses. In this study, SEM and EDX analyses were used to examine the shape, surface characteristics, and elemental composition of Fe nanoparticles. The SEM image in Fig. 6 shows the spherical shape of the nanoparticles, with some minor irregularities. According to the calculations combined to the SEM figure which clearly states, the effective nano particle size is between 8 nm and 16 nm. However, the majority of the nano particles deposited as a thin film on the substrate is between 10 nm and 14 nm. This indicates a high degree of uniformity in particle sizes as the thin layer doesn't show massive particle accumulation.

The aggregated nanoparticles exhibited a rough surface morphology that might be caused by van der Waals forces that attract the atoms and molecules of the particles and hold them together. Such study holds important implications in the field of nanotechnology, as it sheds light on the behavior of nanoparticles in different environments.

**Fig. 7:**
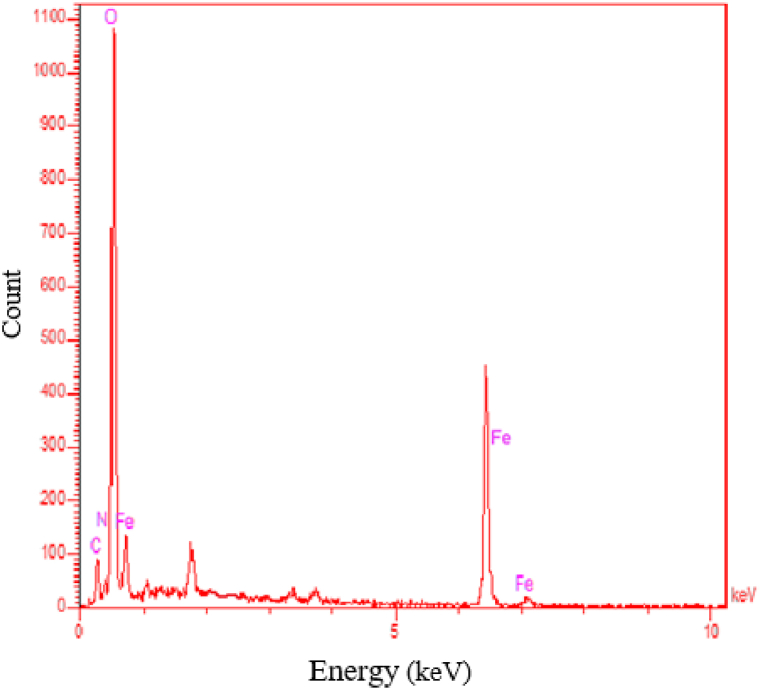
EDX data of the synthesized iron nanoparticles Fe NPs.

Further surface characteristics of the Fe NPs were analyzed by EDX to give information regarding the composition of the elemental nanoparticles. As shown in Fig. 7, EDX analysis results confirmed the element structure of the Fe NPs.

The preparation method allowed for detecting the typical peaks for C, O, and Fe, with their respective weight percent being 12.6 % C, 55 % O, and 32.85 % Fe. It is interesting to note that the peaks coming from carbon and oxygen show that the organic substance from the pomegranate peel extract was absorbed on the surface or in close vicinity to the NPs. This finding informs a lot about the Fe NPs' behavior and their interaction with organic substances. Overall, the SEM and EDX characterization studies support the fact that these Fe NPs could be applied to a wide promising range of utilities, from environment remediation to biomedical and cancer research.

Inclusively, the SEM and EDX studies in this research provided comprehensive knowledge regarding the morphology and elemental makeup of the Fe NPs. These findings will be of importance when the nanoparticles are to be used for different applications.

### 3.2 Germination ratio (GR)

Z3 (seed priming with 30 mM Fe NPs) exhibits a clear and significant change in the percentage of germination as compared to no priming. Thus, 30 mM Fe NPs priming (nano-priming), which affects various growth-related parameters including GR in maize (Fajr-1), which demonstrated significant GR (64.1 %), was the best treatment (Table 1). The same applies to the three salt concentrations S1, S2 and S3. Soaking maize seeds with iron nanoparticles caused an increase in the germination percentage, especially with nano concentration 20 mM, although there were no significant differences between the salt treatments, and this is consistent with earlier studies [28,29]. The level of moisture in seeds may rise due to nanoparticles, which may also trigger radical tissues to begin absorbing water [30,31]. According to Lorrai et al., the germination period enhanced the manufacture of gibberellic acid, increasing the germination ratio [32]. However, only a limited number of processes were certainly suggested: The development of nanopores for enhanced water uptake, the beginning of the reactive oxygen species/antioxidant network in seeds, the production of hydroxyl radicals to weaken cell walls, and the stimulation of amylase to speed up starch digestion are all effects of nano-priming [19]. When NPs reach the seed coat, it produces ROS and hence activates a number of downstream processes [33], which increase the germination ratio.

**Table 1:**
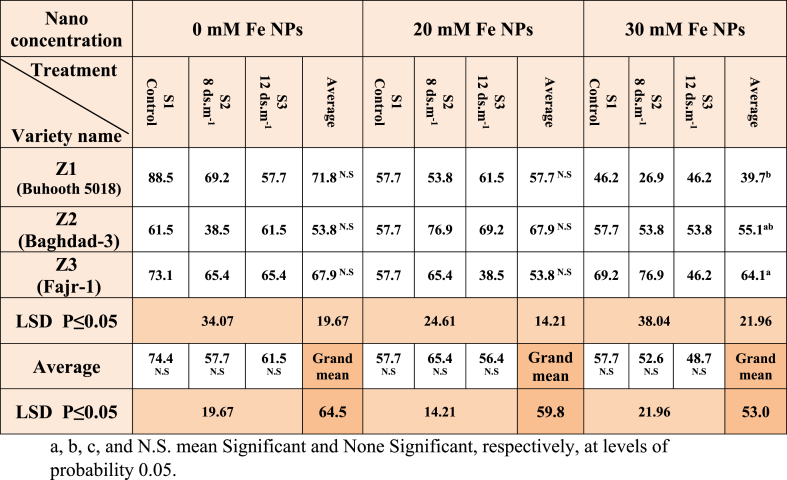
Germination ratio of maize seedlings treated with different salt concentrations.

### 3.3 Seedling vigor index (SVI)

The results of Table 2 indicate that the coefficient of seedling strength for non-nano stimulated plants was distinguished by the Z1(Buhooth 5018) cultivar which recorded 567. As for the salt treatments, the two treatments S2 and S3 were the lowest in the mean of seedling strength was 280 and 359, respectively, while the stimulation of iron NPs concentration of 20 mM was distinguished by the two cultivars Z1 and Z2. Significant differences were recorded gradually between the three treatments S1, S2 and S3. The results were similar for plants stimulated at nano concentration of 30 mM for the cultivars and salinity treatment.

**Table 2:**
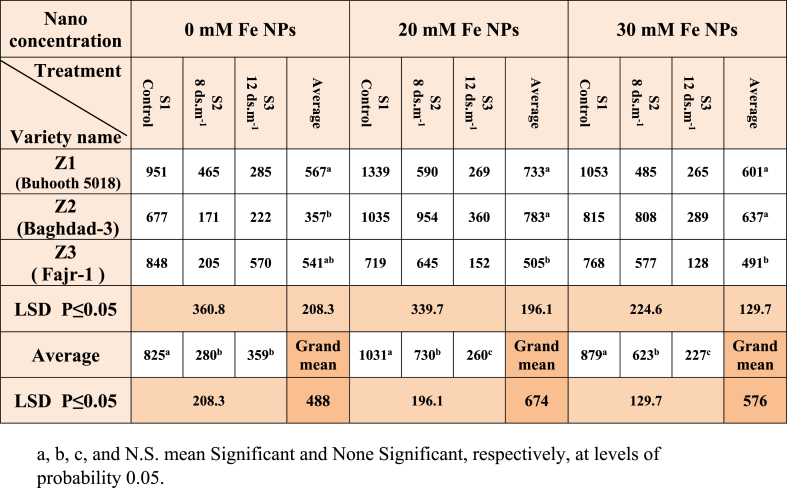
Seedling Vigor Index (SVI) of maize seedlings treated with different salt concentrations.

Nano-priming has proven to be a successful method in improving seed vigor index (SVI) assessment due to the fact that nano-priming enhances the integrity of the seed cell membrane, which results in a higher efficiency of phosphorylation, according to the research conducted by Acharya *at el* [34]. This increased efficiency leads to an improvement in the overall health and productivity of the seed, which can have a significant impact on crop yields. In essence, nano-priming is a promising technique that can be used to optimize seed performance, and thus contribute to the development of sustainable agricultural practices. A new and promising technique for enhancing seed germination, seedling vigor, and development has been identified as seed priming with micronutrient NPs. Seed priming with nanoparticles (NPs) has the potential to enhance the growth of maize crops, even when exposed to high salinity concentrations. This improvement can be attributed to various mechanisms, which include the augmentation of specific enzyme activities such as superoxide dismutase (SOD), catalase (CAT), and phenylalanine ammonia lyase (PAL) [35]. The enhanced permeability of water and nutrients through the seed coat during nano-priming of seeds resulted in the expedited growth, development, and germination of seedlings [36].

### 3.4 Root length (RL)

**Fig. 8:**
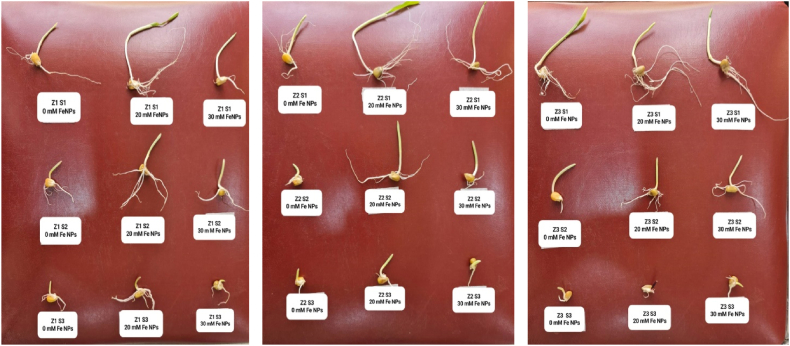
Different Zea mays cultivars Z1 (Buhooth 5018), Z2(Baghdad) and Z3(Fajr-1) and priming with different Fe NPs concentrations (0, 20, 30, mM) and germination on different salinity levels S1 (Control), S2 (8 ds m^−1^) and S3(12 ds m^−1^).

Salinity stress significantly reduced root length (RL) of maize; the highest rate of RL was recorded for the cultivar Fajr-1 at a rate of 3.98 cm, while the control treatment recorded the highest rate of root length with significant differences compared to treatments S2 and S3; the rates were 2.56 and 2.72 respectively (Table 3), (Fig. 8). A significant distinction was observed in all the parameters mentioned above between the control and Fe NPs treated plants. Nano-priming is more effective on RL; the plants stimulated with a concentration of 20 mM of Fe NPs recorded the highest rate of root length in the two cultivars Z1 and Z2 and with significant differences with Fajr-1cultivar plants that recorded 5.19 cm at the time when the treatments S1, S2 and S3 were arranged with the rates that were significant among them. The results were also different in the plants treated with a concentration of 30 mM of Fe NPs, where the distinction was clear with the average RL of the cultivar Fajr-1 (Z3) with significant differences than other two cultivars. While the treatments S2 and S3 recorded a negative decrease compared to the control plants for the same nano concentration.

**Table 3:**
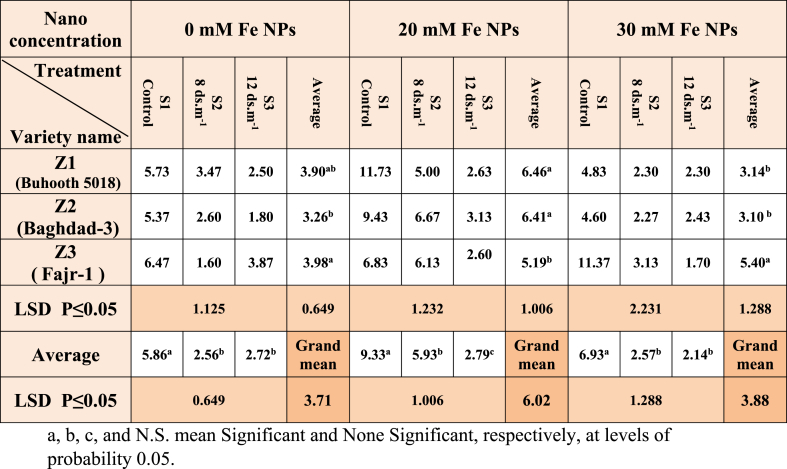
Root length (RL) of maize seedlings treated with different salt concentrations.

Radical length is one of the most important traits for salinity stress due to the contact of roots with soil and their ability to absorb water and minerals from it, so the length of the radical is a basic signal for the response of plants to salinity conditions. Study by Khodarahmpour et al. found that length of radical, plumule, and seedling decreased as salinity levels increased in all hybrid maizes, and the best length of radical and plumule was with control treatment [37]. This has also been proven by Habibi and Aleyasin [38].

### 3.5 Shoot length (SL)

There is a clear significant of the shoot length between the cultivar Fajr-1 (4.02 cm) and the other two cultivars Z1 (2.73) and Z2 (2.77) (Table 4), as the control treatment showed the highest rate of shoot length compared to S2 and S3, which recorded the lowest rates of lengths in unstimulated plants. While the plants treated with Fe NPs at a concentration of 20 mM recorded a clear effect on cultivars Z2 (5.77 cm) and Z1 (5.73 cm) with shoot lengths compared to cultivar Z3 as shown in Fig. 8. Significant differences were arranged between the three treatments S1, S2 and S3, respectively. Nano treatment at a concentration of 30 mM of Fe NPs showed no differences it rose to significance among the three cultivars, while significant differences were recorded between the three salt treatments S1, S2, and S3, respectively.

**Table 4:**
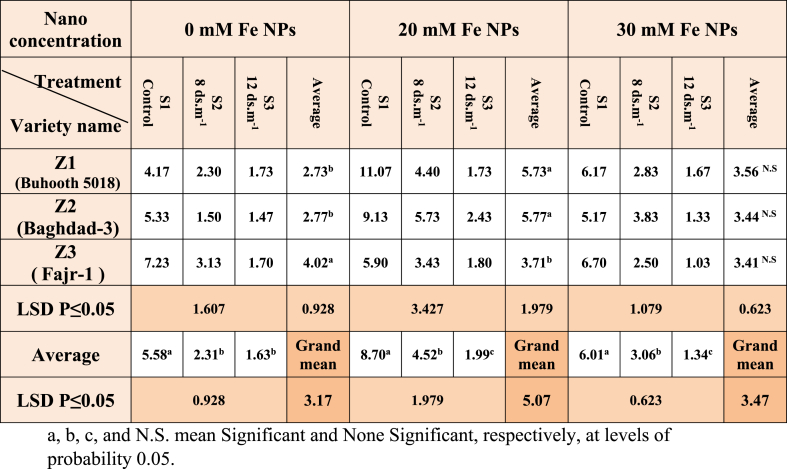
Shoot length (SL) of maize seedlings treated with different salt concentrations.

Shoot length could potentially be attributed to the presence of aquaporins, which possess a significant function in enabling the plant-water interactions. These interactions are particularly important in the processes of water absorption, cell elongation, seed germination, regeneration, and photosynthesis [39]. ROS and aquaporins collaborate in a synergistic manner to enhance the process of seed germination. This phenomenon was observed through the comparison of nano-primed seeds with untreated control seeds and alternative priming techniques [40]. The aquaporin signaling pathway is activated by an increase in ROS, which also affects the phosphorylation sites in essential aquaporin proteins and leads to increased water uptake that led to increase photosynthesis and support growth and elongation [41]. Ghassemi-Golezani et al. [42] recorded the application of nano sized zero-valent iron induces plant growth by overexpressing the CsHA1 gene, triggering plasma membrane H^+^-ATPase, and subsequently enhancing Fe absorption which lead to increase growth. Also, Rajput et al. explained that because nanoparticles are so tiny, they can potentially pass through physiological barriers and penetrate plant tissues [43].

### 3.6 Root length stress tolerance index (RLSTI)

It is inferred from the results of Table 5 that there are no significant differences between the three cultivars, as well as between the salt treatments of unstimulated plants with Fe NPs. While the cultivar Fajr-1 (83.2) in plants stimulated with concentration 20 mM of Fe NPs recorded the highest rate of stress tolerance coefficient in root length. As for the saline treatments, S2 recorded the highest rate (76.5) compared to the salt concentration 12 ds m^−1^ in treatment S3, which recorded 36.6. There was no clear significant between the three cultivars of plants stimulated at a concentration of 30 mM of Fe NPs, as well as the salinity concentrations used. Fe NPs induced stronger rooting growth and expansion than other nano-metal such as Cu and Co NPs [44].

**Table 5:**
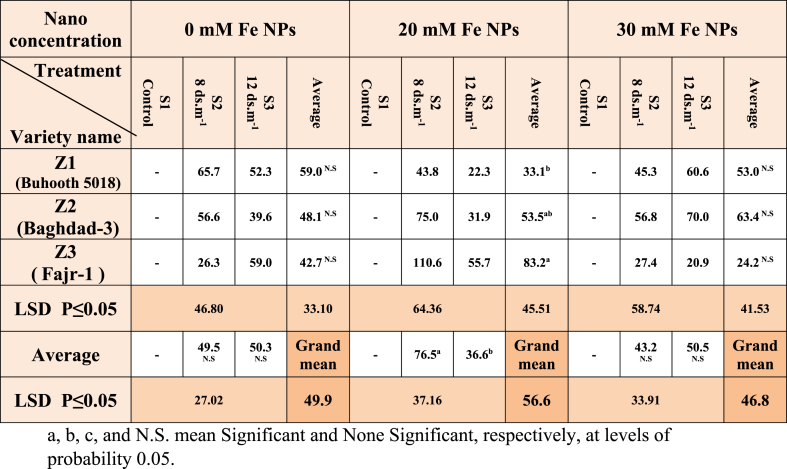
Root length stress tolerance index (RLSTI) of maize seedlings treated with different salt concentrations.

### 3.7 Shoot length stress tolerance index (SLSTI)

In the mean tolerance coefficient for the length of the shoot of the stressed plants (Table 6), the cultivar Z1 was recorded in the non-stimulated plants the highest as cultivar Z2 the lowest, while the differences did not reach significant between the two salt treatments S2 and S3. No significant differences were recorded between the cultivars stimulated with the 20 mM Fe NPs at the time when the saline treatments showed significant differences between the two treatments S2 and S3 and they were 59.5 and 26.4, respectively. In the plants stimulated with the 30 mM nano concentration, Baghdad-3 cultivar had the highest rate of the shoot length stress tolerance index (52.1), and the differences were significant between the two saline treatments S2 and S3. According to Rui et al., Fe_3_O_4_ NPs can serve as an alternative to traditional regular sources of iron for *Arachis hypogaea* plants and provide them with an iron-rich origin [45]. Iron NPs supplementation increased plant height, root length, chlorophyll, and iron levels while regulating antioxidant enzyme and phytohormone activity (reduced ABA and increased GA3 content).

**Table 6:**
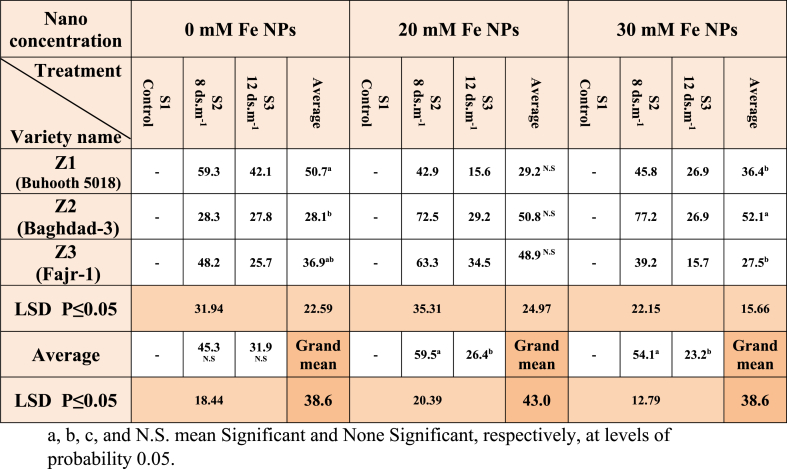
Shoot length stress tolerance index (SLSTI) of maize seedlings treated with different salt concentrations.

### 3.8 Root: shoot dry weight ratio

One of the fundamental features that may be used to evaluate the different cultivars under study's overall physiological state is the root: shoot ratio. It is clear from the results of Table 7 that there are no significant differences between the three cultivars of maize in the percentage of dry weight of roots and shoots in non-stimulated plants, while the salty treatments S3 and S2 of the same plants were characterized by the highest rates of this percentage and were 29.1 and 27.5. The results were similar for plants stimulated with a nano concentration of 20 mM of Fe NPs for the cultivate and salinity treatments. Plants stimulated at a concentration of (30 mM) Fe NPs did not show any significant differences between the cultivars and the salt concentrations.

**Table 7:**
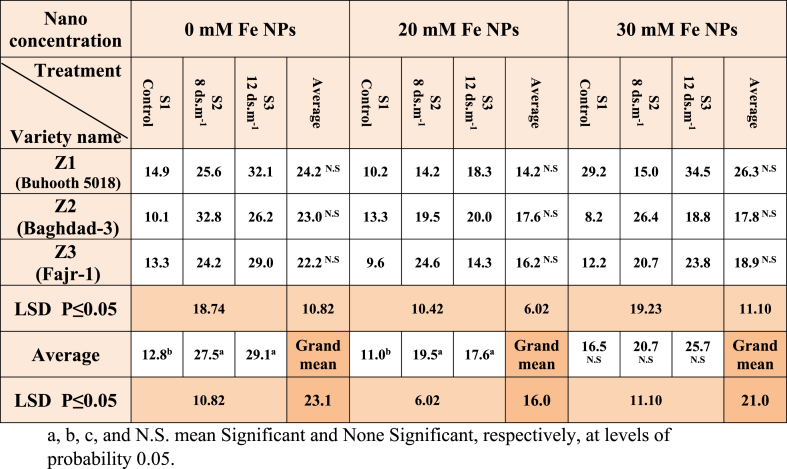
Root: Shoot ratio of maize seedlings treated with different salt concentrations.

Under conditions of high salinity, Na ^+^ might result in a reduction in the movement rate of critical ions like NO_3_, which in turn lowers the N-containing compounds and eventually prevents plant development and biomass formation [46].

Zero-valent metal nanoparticles, such as Cu^0^/Cu^2+^and Fe^0^/Fe^3+^, have been found to enhance the photosynthetic process of plants through electron transfer reactions. The aforementioned reactions give rise to the establishment of an electric field spanning the photosynthetic membrane, concurrently leading to the accumulation of protons within the membrane vesicle. Subsequently, this process serves to transform redox-free energy into an electrochemical potential specific to protons. The energy contained within the previously mentioned proton electrochemical potential is subsequently utilized by a protein complex that resides within the membrane, commonly known as ATP-Synthase, to produce adenosine triphosphate (ATP) from adenosine diphosphate (ADP) through the covalent attachment of a phosphate group [31].As a result of the photosynthetic process, the energy that light initially gave is stored as redox-free energy, which may then be used to reduce carbon dioxide. The conversion of light energy into chemical energy through this mechanism enables plants to manufacture their own food, which is necessary for their survival. Plants can improve their photosynthetic process and ultimately produce more food by utilizing zero-valent metal nanoparticles with suitable redox potential energy. This could have significant implications for agriculture and the production of crops, allowing for a more sustainable and efficient food production system [44].

Nano formulation of fertilizers has emerged as a promising alternative to traditional chemical fertilizers. In comparison to chemical fertilizers, studies have shown that the application of nanoiron chelate and nanozinc can greatly increase the levels of phosphorus, biomass, crude protein, and soluble sugar contents in *Zea mays* L [21,47].

## 4 Conclusion

Natural products contain phytochemical compounds that serve as the main reducing and stabilizing agents in the synthesis of (Fe NPs) nanoparticles. The creation of iron nanoparticles was made possible by the use of PPE, a readily available fruit waste *(Punica granatum*). The FTIR results indicate the formation of functional groups in iron nanoparticles, while the UV–visible absorbance shows UV absorption peak existence at around 301 nm. To characterize the morphological features of iron nanoparticles, higher-resolution observations were made using SEM. Spherical iron nanoparticles were produced at a size range of 8.7–17.8 nm, and elemental separation was achieved through EDX results, indicating the weight percentage of (Fe NPs) nanoparticle. The results indicated that the synthesized Fe NPs exhibited a high level of stability, with a value of 5.5 mV.

The effects of NaCl, salinity stress on the maize seed, and (Fe NPs) nanoparticles treatment were compared. Salinity was created using NaCl for (0, 8, 12 ds m^−1^) and (Fe NPs) nanoparticle treatment concentrations were 20 mM and 30 mM for 7 days in the lab. The growth parameters were measured. Especially at a concentration of 20 mM, which had a clear effect on increasing the rates of SVI, RL, SL, RLSTI, and SLSTI when compared to plants that were not soaked with Fe NPs. It can be concluded that green-synthesized Fe-NP enhances *Zea* Mays germination under saline conditions to a greater extent compared to Fe-NP synthesized via conventional methods as well as commercial Fe.

## CRediT authorship contribution statement

**Hiba Fouad Abdulfatah:** Writing – original draft, Software, Methodology, Formal analysis. **May Fahmi Abdulrahman:** Writing – review & editing, Methodology, Data curation. **Enas Fahd Naji:** Investigation, Data curation.

## Data availability statement

The data that support the findings of this study are available from the corresponding author upon reasonable request.

## Declaration of competing interest

The authors declare that they have no known competing financial interests or personal relationships that could have appeared to influence the work reported in this paper.
